# Innate Immunity in the Pathogenesis of Selected Autoimmune Neurological Diseases

**DOI:** 10.3390/jcm14207235

**Published:** 2025-10-14

**Authors:** Julia Rudnicka-Czerwiec, Halina Bartosik-Psujek

**Affiliations:** Department of Neurology, Faculty of Medicine, University of Rzeszów, al. Tadeusza Rejtana 16C, 35-959 Rzeszów, Poland

**Keywords:** innate immunity, innate immune response, MS, NMOSD, MOGAD, MG, CIDP

## Abstract

The human immune system consists of two main components: innate and adaptive immunity. To date, research on the pathogenesis of autoimmune neurological diseases has primarily focused on the role of adaptive immunity. However, growing evidence highlights the significant contribution of innate immune mechanisms in the development of neurological disorders. The aim of this article is to present the current state of knowledge regarding the involvement of innate immunity in the pathogenesis and treatment of selected autoimmune neurological diseases: multiple sclerosis (MS), neuromyelitis optica spectrum disorder (NMOSD), MOG antibody-associated disease (MOGAD), myasthenia gravis (MG), and chronic inflammatory demyelinating polyneuropathy (CIDP). A literature review was conducted, including both experimental and clinical data on the activity of innate immune effector cells—such as dendritic cells, macrophages, microglia, and natural killer (NK) cells—as well as plasma proteins, including the complement system. Relevant clinical and preclinical studies on targeted therapies affecting these components were also identified. All analyzed diseases demonstrate the involvement of innate immune elements in the initiation and maintenance of the inflammatory process. Furthermore, it has been shown that therapies targeting these components may offer clinical benefits.

## 1. Introduction

The human immune system is composed of two major components: innate (nonspecific) and adaptive (specific) immunity. Innate immunity relies on the rapid activation of nonspecific defense mechanisms and the presence of natural barriers within the body. In contrast, adaptive immunity develops as a targeted response against microorganisms or antigens previously recognized by innate immune mechanisms [1].

Innate immunity constitutes the first line of defense against infectious agents. Its mechanisms act rapidly—within minutes to hours after pathogen exposure—whereas adaptive immunity requires a longer period for activation. The primary functions of innate immunity are to prevent infection, eliminate pathogens, and initiate as well as modulate the adaptive immune response.

Key components of innate immunity include physical barriers (the skin and mucous membranes); effector cells such as granulocytes, monocytes, macrophages, dendritic cells (DCs), natural killer (NK) cells, and innate lymphoid cells (ILCs); as well as epithelial and endothelial cells. Plasma proteins—including elements of the complement cascade—and cellular receptors that recognize molecular patterns also play critical roles [2,3,4,5].

Growing evidence supports the immunological basis of numerous neurological disorders, establishing neuroimmunology as one of the most rapidly advancing fields of neurology [6].

The aim of this paper is to review the current state of knowledge on the role of innate immunity in the pathogenesis of selected immune-mediated neurological disorders and to evaluate the therapeutic potential of its components. The analysis focuses on diseases chosen by the authors including Multiple Sclerosis (MS), Neuromyelitis Optica Spectrum Disorders (NMOSD), Myelin Oligodendrocyte Glycoprotein Antibody-Associated Disease (MOGAD), Myasthenia Gravis (MG), and Chronic Inflammatory Demyelinating Polyradiculoneuropathy (CIDP). In this review major autoimmune neurological diseases were selected to illustrate pathology of the central nervous system, the neuromuscular junction, and the peripheral nervous system. This choice allows to provide a broad perspective across different levels of the nervous system.

## 2. The Role of Innate Immunity in the Pathogenesis of Neurological Diseases

### 2.1. Multiple Sclerosis

MS is the most common autoimmune demyelinating disorder of the central nervous system (CNS), although its pathogenesis remains incompletely elucidated [7]. The role of the immune system in MS development is indisputable and has been intensively investigated for decades. While early research focused primarily on adaptive immunity, including B and T cells and autoantibodies, it is now evident that innate immune elements also play crucial roles in disease pathogenesis [8,9].

#### 2.1.1. Dendritic Cells

Compelling evidence implicates DCs in MS [10]. In the murine model Experimental Autoimmune Encephalomyelitis (EAE), Langerhans cells—specialized skin DCs—migrate to lymph nodes after recognizing myelin antigens, where they present them to T lymphocytes. This signal alone is sufficient to initiate EAE [11]. DCs are present in multiple CNS compartments, including the cerebrospinal fluid (CSF), choroid plexus, meninges, and perivascular spaces [12]. Within the CNS, their primary pathogenic role is to enhance antigen presentation to T cells, facilitating their transmigration across the blood–brain barrier (BBB) and subsequent invasion of neural tissue [13]. DCs drive polarization of T cells toward a pro-inflammatory Th17 phenotype, characterized by secretion of interleukin-17 (IL-17) and granulocyte–macrophage colony-stimulating factor (GM-CSF) [14]. Conversely, tolerogenic DCs (TolDCs) can induce protective effects, promoting T-cell anergy or differentiation into regulatory T cells (Tregs) [15]. Tregs generated under TolDC influence exhibit increased expression of cytotoxic T-cell antigen-4 (CTLA-4), thereby limiting excessive T-cell activity. They also lose the ability to produce pro-inflammatory cytokines such as interferon-gamma (IFN-γ) and interleukin-2 (IL-2) [16]. In MS patients, DCs are present in both demyelinating plaques and meninges, where pro-inflammatory phenotypes predominate [17].

#### 2.1.2. Macrophages

Macrophages are the most abundant immune cells in demyelinating plaques. These include resident microglia and macrophages derived from infiltrating peripheral monocytes, which normally are absent in CNS and require specific activation signals to cross the BBB [18,19,20]. EAE studies demonstrate that microglia are essential for disease initiation, whereas activation of peripheral monocytes drives disease progression. Experimental depletion of monocytes prior to symptom onset delays disease development and reduces severity, while depletion after clinical onset halts progression [21,22]. Elevated peripheral monocyte counts at disease onset correlate with faster progression and accelerated disability accumulation [23]. Once within the CNS, monocytes differentiate into DCs or macrophages. Depending on their polarization, macrophages acquire either a pro-inflammatory (M1) or anti-inflammatory (M2) phenotype [24]. Pro-inflammatory polarization, driven by cytokines such as GM-CSF, IFN-γ, and tumor necrosis factor-alpha (TNF-α), predominates during the early stages of EAE and during relapses, causing both direct and indirect neural damage [25,26,27]. In contrast, M2 macrophages are more prominent during remission and promote tissue repair [28,29].

#### 2.1.3. Microglia

Microglia play a central role in MS pathology, contributing to myelin phagocytosis, antigen presentation, and the release of pro-inflammatory cytokines within active lesions [30]. In EAE, microglial inactivation delays disease onset and attenuates clinical severity [31]. Histopathological analyses of human MS tissue and EAE models confirm their involvement: in early active lesions, microglia account for approximately 40% of phagocytes, localize centrally, and exhibit a pro-inflammatory phenotype, whereas in inactive plaques they shift toward anti-inflammatory states [32]. In animal models, myelin internalization induces a regenerative microglial phenotype that promotes oligodendrocyte differentiation and supports remyelination [33,34,35,36]. In progressive MS, microglia within chronic plaques become reactivated, accumulate at lesion margins, and drive gradual lesion expansion [30]. Transcriptomic studies further demonstrate microglial alterations beyond visible plaques, including upregulation of lipid metabolism-related genes in normal-appearing white matter and iron metabolism-related genes in gray matter [37,38].

#### 2.1.4. Astrocytes

Astrocytes have long been implicated in the pathogenesis of MS, with their presence in lesions recognized as early as the 19th century. In EAE, depletion of reactive astrocytes during the acute phase exacerbates clinical symptoms and CNS inflammation [39,40], whereas selective depletion during the chronic phase ameliorates disease and suppresses the recruitment of microglia and monocytes [41]. Astrocytes are activated by pro-inflammatory cytokines and pathogen-associated molecular patterns (PAMPs) [42,43]. Once activated, they produce a broad spectrum of chemokines that attract leukocytes to perivascular and parenchymal compartments [44,45,46], including CXCL12, which facilitates the recruitment of monocytes, T cells, B cells, and plasma cells into active and chronic plaques [47]. Moreover, astrocytes limit T-cell infiltration into the CNS by expressing pro-apoptotic molecules such as Fas ligand (FASL) and TNF-related apoptosis-inducing ligand (TRAIL) [48].

#### 2.1.5. NK Cells

The role of NK cells in MS is complex and remains unclear. Evidence supports both protective/regulatory functions—limiting autoimmunity—and pathogenic effects—enhancing immune responses and promoting relapses [49]. CD56bright NK cells can distinguish activated from resting T cells and selectively eliminate activated T cells through granzyme release or TRAIL-dependent cytotoxicity [50,51,52]. CD56bright NK cells producing granzyme K are enriched in periventricular regions and demyelinating plaques, migrating via the choroid plexus in response to activated T-cell signals [53,54]. Granzyme K release induces mitochondrial dysfunction and oxidative stress in activated T cells, ultimately leading to apoptosis [55,56]. In contrast, CD56dim NK cells mediate antibody-dependent cellular cytotoxicity (ADCC) against both activated and resting T cells [57]. Increased NK-cell tolerance toward T cells, as well as T-cell resistance to NK-cell activity, may accelerate disease progression [50]. Recently identified CD8+ NK-cell subset has been associated with reduced relapse risk [58]. EAE models further confirm the importance of NK cells: their depletion exacerbates disease, whereas their expansion alleviates symptoms [59,60]. NK cells can also exert indirect effects by eliminating activated microglia, thereby limiting Th17 activation [60], and by producing IFN-γ in the meninges, which promotes an anti-inflammatory astrocyte phenotype and induces TRAIL-dependent T-cell apoptosis [61]. Conversely, other studies have implicated NK cells in cortical demyelination, where CD56dim NK cells accumulate near vessels, infiltrate demyelinated gray matter, and mediate perivascular demyelination via ADCC [62]. NK cells have also been reported to impair neurogenesis and neural tissue repair in chronic MS [63,64].

#### 2.1.6. Complement System

Complement activation contributes to all MS subtypes [65,66]. Deposits of C1q, C3d, and C5b-9 complexes are consistently observed in white matter and demyelinating lesions [67,68]. Genetic silencing of early complement activation, particularly at C3, ameliorates EAE severity [69]. Human genetic studies further implicate the C3 variant rs2230199 in white and gray matter damage and cognitive dysfunction [70]. Although the precise cellular targets of complement-mediated injury remain unclear, complement activation markers may identify patients who benefit most from plasmapheresis during MS relapses [71]. Cerebrospinal fluid C3a levels in patients with clinically isolated syndrome and newly diagnosed relapsing–remitting MS may serve as a promising prognostic marker of disease activity—correlating with the emergence of new focal lesions and with the “No Evidence of Disease Activity-3” (NEDA-3) status [72].

### 2.2. Neuromyelitis Optica Spectrum Disorders

NMOSD is an inflammatory demyelinating disorder of the CNS. Approximately 80% of patients are seropositive for immunoglobulin G (IgG) directed against aquaporin-4 (AQP4), a key water channel protein located on the perivascular endfeet of astrocytes [73]. Although the presence of autoantibodies indicates the involvement of adaptive immunity, their pathogenic effects are largely mediated by innate immune mechanisms. AQP4-specific IgG (AQP4-IgG) initiate complement-dependent cytotoxicity (CDC) and antibody-dependent cellular cytotoxicity (ADCC), ultimately leading to astrocyte injury, secondary oligodendrocyte degeneration, and demyelination [74].

#### 2.2.1. Complement System

Binding of AQP4-IgG to AQP4 on the astrocyte surface activates the classical complement pathway. This process begins with C1q binding to the Fc region of IgG within antigen–antibody complexes and progresses through the complement cascade to form the membrane attack complex (MAC), which damages astrocytic membranes [75]. Complement activation also generates C3a and C5a, which increase vascular permeability and create chemotactic gradients that facilitate leukocyte migration across the BBB [76]. Histopathological evidence underscores the role of complement in NMOSD: active lesions exhibit marked perivascular deposition of immunoglobulins and the C9neo antigen, a residual marker of MAC, accompanied by fibrosis and hyalinization of vessel walls [77]. Notably, the absence of complement activation does not necessarily protect against neural injury. In such cases, astrocyte membranes remain structurally intact, but AQP4 undergoes endocytosis with concurrent loss of the sodium-dependent glutamate transporter excitatory amino acid transporter 2 (EAAT2). Disrupted glutamate homeostasis may cause excitotoxicity, neuronal death, oligodendrocyte dysfunction and secondary demyelination [78].

#### 2.2.2. NK Cells

NK cells are key effectors of ADCC in NMOSD. By binding the Fc fragment of AQP4-IgG through FcγRIII (CD16), NK cells become activated and degranulate, releasing perforin and granzymes that lyse astrocytes and drive neurodegeneration and demyelination. Their role has been confirmed in animal models, where blockade of FcγR–antibody interactions significantly reduced astrocytic damage [79]. Increasing evidence also suggests a contribution of other innate lymphoid cells (ILCs) to NMOSD pathogenesis. Type 2 ILCs have been shown to exert protective effects and suppress disease development [80].

#### 2.2.3. Neutrophils

The involvement of neutrophils in NMOSD is supported by both clinical and experimental findings. During relapses, elevated neutrophil counts in CSF are observed in approximately 60% of untreated patients, compared with only 20% during remission [81]. Plasma from NMOSD patients shows increased concentrations of CXCL5 and CXCL8—potent neutrophil chemoattractants—as well as neutrophil elastase (NE) [82]. In murine models, neutrophil depletion attenuated neural tissue injury, whereas increased neutrophil number exacerbated inflammation. Immunohistochemistry revealed degranulated neutrophils within inflammatory foci, suggesting pathogenic activity through NE-dependent mechanisms. Administration of the NE inhibitor sivelestat ameliorated disease severity [83].

#### 2.2.4. Eosinophils

A hallmark of active NMOSD lesions in the spinal cord is intense infiltration of eosinophils within perivascular and meningeal spaces, along with expression of CCR3—the principal receptor for eotaxin, a potent eosinophil chemoattractant [77]. CSF from NMOSD patients contains higher levels of eotaxin-2 and eotaxin-3 compared with both healthy controls and MS patients. Moreover, stimulation of CSF cells with myelin oligodendrocyte glycoprotein (MOG) antigen enhances production of interleukin-5 (IL-5), which recruits and activates eosinophils [84]. Activated eosinophils release cytotoxic proteins including eosinophil cationic protein (ECP), eosinophil-derived neurotoxin (EDN), eosinophil peroxidase (EPX), and major basic protein (MBP). Eosinophils can therefore damage neural tissue through both lytic granule degranulation and ADCC [85].

#### 2.2.5. Microglia

Microglial activation and macrophage infiltration also occur in regions of high AQP4 expression [86]. In animal models, AQP4-IgG induce astrocytic production of complement component C3 [87]. Microglia, which express receptors for C3a, become activated in response to elevated C3 levels [88]. Activated microglia produce C1q, which may promote axonal damage and neurodegeneration independently of full complement activation [89,90]. Conversely, microglia can also provide neuroprotection by phagocytosing myelin debris and supporting remyelination through tissue clearance and regeneration [91,92]. The triggering receptor expressed on myeloid cells 2 (TREM2) plays a pivotal role in this process, enhancing microglial phagocytic activity and degradation of myelin remnants, thereby facilitating neuroprotection in NMOSD-associated demyelination [93].

### 2.3. Myelin Oligodendrocyte Glycoprotein Antibody-Associated Disease

MOGAD is an inflammatory demyelinating disease that most commonly presents as acute disseminated encephalomyelitis, optic neuritis or transverse myelitis. Diagnosis relies on the detection of IgG autoantibodies against myelin oligodendrocyte glycoprotein (MOG), an accessible autoantigen located on the surface of myelin sheaths and oligodendrocyte processes within the CNS [94].

As in NMOSD, autoantibodies are central to pathogenesis, but tissue injury is mediated primarily by effector mechanisms of innate immunity. Demyelination induced by MOG-specific IgG (MOG-IgG) occurs through CDC, ADCC, antibody-dependent cellular phagocytosis (ADCP), as well as by direct disruption of the oligodendrocyte cytoskeleton. Despite increasing research, the immunological mechanisms underlying MOGAD remain incompletely defined [95].

#### Complement System

Among the best-characterized effector processes is complement activation. Its role is supported by preclinical studies [96] and histopathological evidence showing deposition of complement components within demyelinating lesions [97,98,99]. Furthermore, serum from MOGAD patients exhibits greater activity of both the classical and alternative complement pathways compared with healthy controls, as assessed using a multiplex chemiluminescence-based ELISA assay [100]. However, complement activation by MOG-IgG appears less efficient than that induced by AQP4-IgG, likely because MOG-IgG in most patients are predominantly bivalent, a form considered less effective at initiating the complement cascade [101].

### 2.4. Myasthenia Gravis

MG is an autoimmune disorder characterized by impaired neuromuscular transmission. It is highly heterogeneous, both pathogenetically—reflected by diverse autoantibody profiles—and clinically. Antibodies against the acetylcholine receptor (AChR) are the most common autoantibody type, detected in approximately 85% of patients with the generalized form of the disease [102,103].

In AChR antibody–positive MG, aberrant adaptive immune activation leads to pathogenic antibody production through B-cell somatic hypermutation and antigen selection [104]. However, the initiation and maintenance of autoimmunity largely depend on innate immune mechanisms [105,106].

Innate immunity contributes to both the initiation of autoimmunization and the effector phase of MG pathogenesis. Evidence implicates Toll-like receptor (TLR) signaling pathways, the classical complement cascade, and innate immune cells including NK cells, NKT cells, and DCs.

#### 2.4.1. TLRs

Pathogen exposure can trigger aberrant TLR activation in the thymus [107]. Poliovirus (PV), for example, activates the TLR4 pathway, leading to increased expression of chemokines that attract DCs and to cytokine release by Th17 cells, thereby disturbing the balance between effector and regulatory T cells [108,109]. Epstein–Barr virus (EBV) interacts with TLR3 or TLR7, inducing excessive production of pro-inflammatory cytokines such as interferon-beta (IFN-β) and chemokines that recruit peripheral B cells and Th17 cells, which promote germinal center (GC) formation in the thymus [110,111,112,113]. Type I interferons (IFN-I) are pivotal in MG pathogenesis. IFN-β stimulates thymic epithelial cells (TECs) to express AChR, which are subsequently internalized by antigen-presenting cells (APCs), initiating autosensitization to AChR and driving autoantibody production [114].

#### 2.4.2. Complement System

Anti-AChR antibodies exert their pathogenicity through complement activation. The classical pathway is triggered by antibody binding to C1q, leading to assembly of the C4b2a complex (C3 convertase), cleavage of C3, and subsequent formation of the C5 convertase. Generation of C5b initiates formation of the membrane attack complex (MAC), resulting in destruction of the postsynaptic membrane at the neuromuscular junction (NMJ) [115].

#### 2.4.3. NK Cells

NK cells are involved in MG pathogenesis, but their role may be protective or pathogenic depending on phenotype and function [116]. The chemokine receptor CXCR5 promotes migration of follicular helper T cells (Tfh) and B cells into GCs [117]. Based on CXCR5 expression, NK cells can be divided into CXCR5(−) and CXCR5(+) subsets. Transfer of CXCR5(−) NK cells into rats with experimental autoimmune myasthenia gravis (EAMG) alleviated disease symptoms, reduced Tfh frequencies, and lowered anti-AChR antibody titers [118]. Conversely, NK cells promote Th1 activation by enhancing IFN-γ secretion and inhibiting transforming growth factor-β1 (TGF-β1), thereby contributing to EAMG development. Mice deficient in NK cells display reduced anti-AChR antibody levels, impaired Th1 responses, and resistance to EAMG. In MG patients undergoing plasmapheresis, responders exhibited significantly reduced NK-cell cytotoxicity [119].

#### 2.4.4. NKT Cells

NKT cells modulate immune responses by producing immunoregulatory cytokines. Activation of NKT cells with their agonist α-galactosylceramide (α-GalCer) enhances IL-2 production, a key driver of Treg development [120]. In mice treated with α-GalCer, Tregs were not only more abundant but also functionally more potent, expressing higher levels of anti-apoptotic proteins Foxp3 and Bcl-2. Consequently, NKT cells protected against EAMG induction [121]. DCs are also central APCs. In vivo inhibition of DC maturation reduced IL-1β secretion, lowered Tfh-cell numbers, and ameliorated EAMG severity [122].

### 2.5. Chronic Inflammatory Demyelinating Polyneuropathy

CIDP is an autoimmune disorder characterized by chronic inflammation and damage to the myelin sheaths and axons of peripheral nerves [123]. Growing evidence indicates that autoantibodies contribute significantly to demyelination in CIDP by injuring myelin and axons, disrupting Schwann-cell membranes and damaging the nodes of Ranvier [124]. Innate immune mechanisms are also critical in CIDP pathogenesis.

#### 2.5.1. Complement System

Their involvement of complement system is supported by the detection of complement component deposits in the myelin sheaths of sural nerves in patients [125,126] and by elevated levels of activated complement components (C3d) in serum during active disease [127]. Lewis rats are a standard animal model of experimental autoimmune neuritis (EAN), and studies in these models have demonstrated that complement inhibition can restore nerve function and slow disease progression [128,129,130]. The importance of complement in CIDP is further underscored by genetic studies: cases of early-onset neuropathy resembling CIDP have been described in children carrying rare mutations in the CD59 gene, which encodes a complement regulator. These mutations resulted in excessive complement activation, suggesting that complement dysregulation may represent a key pathogenetic factor in CIDP [131]. More recently, an in vitro “human-on-a-chip” functional model showed that serum from CIDP patients induced binding of autoantibodies to Schwann cells and motor neurons, accompanied by deposition of C3b and C5b-9 [132].

#### 2.5.2. Macrophages

Macrophages also play a pivotal role in the pathogenesis of inflammatory demyelinating polyneuropathies such as Guillain–Barré syndrome (GBS) and CIDP [133,134]. One mechanism initiating their activation may be molecular mimicry, in which pathogen epitopes resemble peripheral nerve antigens [135]. Alternatively, disease may begin with activation of resident macrophages, which acting as APCs, erroneously recognize myelin structures [136]. Once the inflammatory cascade is triggered, peripheral blood monocytes—guided by adhesion molecules such as selectins and ICAM-1—migrate into the endoneurium [137,138]. There, under the influence of matrix metalloproteinases, they differentiate into macrophages [139]. Both resident and monocyte-derived macrophages then amplify inflammation through cytokine secretion. In addition, macrophages directly damage myelin by releasing proteolytic enzymes and phagocytosing myelin structures [140,141,142].

#### 2.5.3. DCs and NK Cells

DCs and NK cells have also been proposed to contribute to CIDP pathogenesis. However, beyond observed alterations in their levels following immunoglobulin therapy, there is currently no conclusive evidence confirming their substantial involvement in the disease process [143,144].

**Figure 1:**
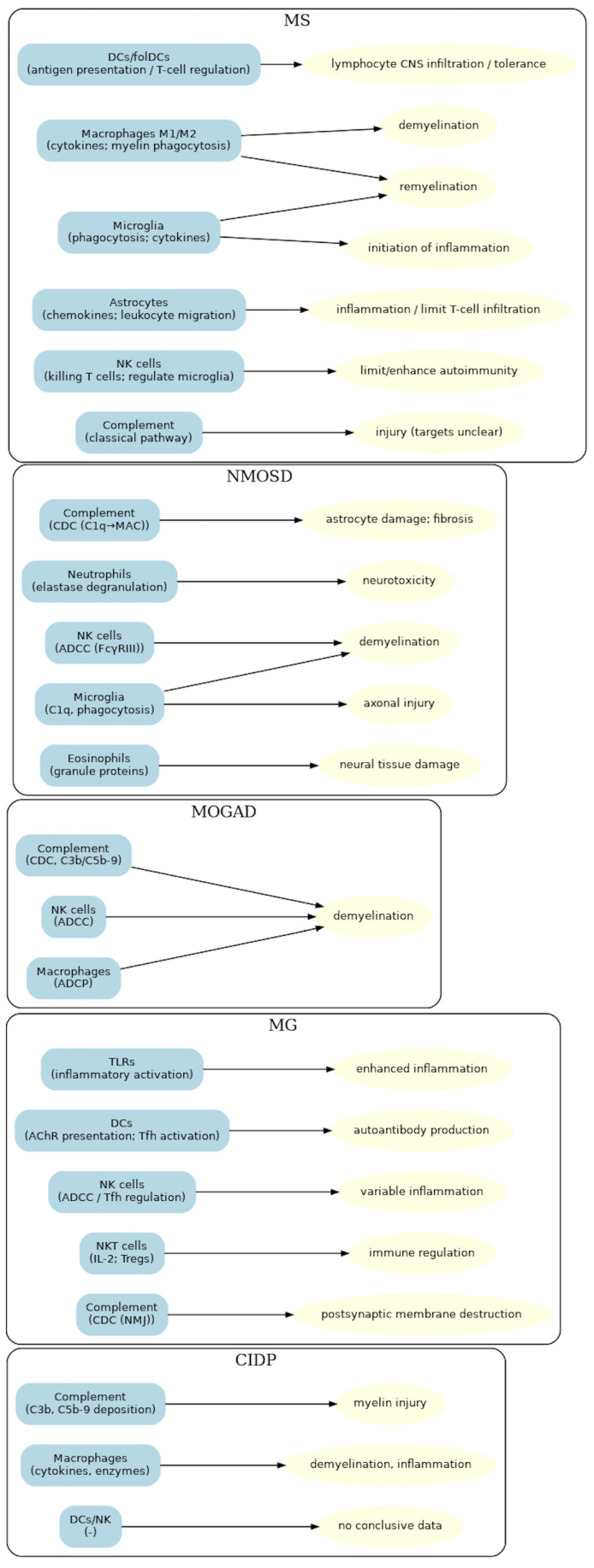
Links between innate immune elements, their mechanisms of action and pathogenic effects in neurological diseases.

The connections between innate immune elements, their mechanisms of action and pathogenic effects in neurological diseases are summarized in Figure 1.

## 3. Elements of Innate Immunity as Potential Therapeutic Targets

### 3.1. Treatment of MS

Current therapeutic strategies for MS focus on managing relapses with glucocorticoids and reducing inflammatory activity with disease-modifying therapies (DMTs). These agents alter the disease course primarily by suppressing or modulating immune responses. Currently approved and widely used DMTs predominantly target adaptive immunity, although they may indirectly influence innate immune pathways [145]. Increasing evidence supports the feasibility of developing therapies that directly target components of innate immunity.

Promising results have been reported with DCs–directed therapies. Agents that inhibit DC maturation toward a pro-inflammatory phenotype include cytokine inhibitors such as anakinra and tocilizumab [146], MOR103 [147,148], KB003, and BVDU [149], as well as T-cell costimulation inhibitors, including CTLA4-Ig, a cytotoxic T-lymphocyte antigen-4 fusion protein. These agents act by suppressing immunogenic DC functions or by inducing a tolerogenic phenotype. Research efforts also include recombinant chimeric antibodies such as anti-DEC205-MOG [150] and anti-CD11c-MOG [151], which deliver tolerogenic antigens to DCs through specific receptors [152,153,154,155,156,157]. Another approach involves the use of ex vivo-generated TolDCs [158]. This strategy is constrained by technical challenges related to isolating, purifying and culturing autologous DCs or their precursors, which increases cost and complexity [159]. As an alternative, nanoparticles carrying antigens and immunomodulators have been designed to induce TolDCs in vivo [160]. An additional therapeutic approach involves blocking DCs migration from inflamed tissues to peripheral lymphoid organs. Arc/Arg3.1, a cytoskeleton-regulating protein, plays a critical role in DCs motility and T-cell activation in both EAE and allergic dermatitis models. Targeting Arc/Arg3.1 may enable selective modulation of immune responses [161].

Microglia have also emerged as promising therapeutic target in MS. Inhibition of microglial activity with the orally administered colony-stimulating factor 1 receptor (CSF1R) inhibitor PLX5622 significantly reduced both clinical symptoms and pathological features in EAE. The treatment created a regenerative microenvironment by selectively enriching lesion sites with anti-inflammatory microglia and mature oligodendrocytes. PLX5622 is currently under evaluation in preclinical studies [162].

Another experimental strategy is the PADRE-Kv1.3 vaccine, developed to modulate immune responses by inducing antibodies against the Kv1.3 potassium channel, which is critical for the activity of microglia and macrophages. In a 2018 study, PADRE-Kv1.3 significantly ameliorated clinical symptoms and reduced CNS pathology in EAE. Treatment diminished microglial and macrophage infiltration and promoted a shift toward the anti-inflammatory M2 phenotype. However, studies on PADRE-Kv1.3 remain limited to animal model and clinical data in humans regarding efficacy and safety are currently lacking [163].

### 3.2. Treatment of NMOSD

Therapeutic management of NMOSD includes both the treatment of acute relapses and long-term relapse prevention. Acute attacks are typically managed with high-dose intravenous glucocorticoids, while therapeutic plasmapheresis is reserved for refractory or severe cases. Intravenous immunoglobulins (IVIG) may be considered in selected clinical contexts. Preventive strategies include not only classical immunosuppressants but also biological agents targeting both adaptive and innate immune pathways. Adaptive immunity-directed therapies include satralizumab and tocilizumab, which block the interleukin-6 receptor (IL-6R), as well as inebilizumab and rituximab, which deplete B cells. The principal innate immune target is the complement system, whose activation plays a pivotal role in NMOSD pathogenesis [164,165].

Eculizumab and ravulizumab are monoclonal antibodies that inhibit complement component C5, thereby preventing formation of MAC. In a phase 3 clinical trial, eculizumab reduced relapse risk by 94% in AQP4-IgG-positive patients [166], an effect confirmed in open-label extension [167] and monotherapy studies [168], independent of demographic and clinical variables [169]. Ravulizumab, a longer-acting analogue, reduced relapse risk by 98.6% in clinical trials [170]. Both agents demonstrated favorable safety profiles [166,167,170].

There is also a case report suggesting potential benefit from adjunctive cetirizine in NMOSD, with a proposed mechanism involving inhibition of eosinophil activation and function. However, the authors emphasized that these findings constitute class IV evidence, and no clinical trials of cetirizine in NMOSD are currently ongoing [171].

### 3.3. Treatment of MOGAD

There is currently no disease-specific therapy for MOGAD. Management like NMOSD involves both relapse treatment and relapse prevention. High-dose intravenous methylprednisolone remains the first-line therapy for acute relapses. In cases of incomplete neurological recovery, IVIG or plasmapheresis may be effective. Maintenance therapies include mycophenolate mofetil, azathioprine, IVIG, oral glucocorticoids, rituximab, and interleukin-6 receptor antagonists such as satralizumab [172]. At present, no clinical trials are evaluating therapies that directly target elements of innate immunity in MOGAD.

### 3.4. Treatment of MG

Standard therapy for MG consists of acetylcholinesterase inhibitors and immunosuppressive agents. However, approximately 10% of patients remain refractory to this treatment [173].

Advances in understanding the role of innate immunity in MG pathogenesis have prompted the development of novel therapeutic strategies. Anifrolumab, a monoclonal antibody targeting the IFN-I receptor, has been approved for systemic lupus erythematosus (SLE)—an autoimmune disease characterized by excessive IFN-I production [174,175]. Although no clinical trials are currently investigating IFN-I blockade in MG, modulation of this pathway may represent a promising future strategy.

TLR antagonists are also being explored as potential therapies for autoimmune diseases due to their ability to suppress inflammatory signaling and cytokine induction [176]. Chaperonin 10 exhibits anti-inflammatory effects by inhibiting TLR4 activation, as demonstrated in clinical studies in rheumatoid arthritis (RA) [176]. M5049, a TLR7/8 antagonist, protected against SLE development in preclinical murine models and was shown to be safe in a phase I trial in healthy volunteers [177,178]. IMO-3100, a dual TLR7/TLR9 antagonist, demonstrated therapeutic potential in autoimmune disease by reducing skin inflammation in a phase 2a trial in psoriasis [179]. Despite their potential, no clinical studies are currently evaluating TLR inhibitors in MG.

In recent years, the complement system has attracted the greatest attention in the development of novel MG therapies. Eculizumab, a high-affinity monoclonal antibody against complement component C5, inhibits C5 convertase activity and prevents MAC formation. Clinical trials have demonstrated that eculizumab reduces the risk of exacerbations and improves daily functioning and quality of life in treatment-refractory, AChR-antibody-positive MG [180,181,182]. Real-world studies further support its favorable safety profile and effectiveness in reducing exacerbations, while enabling safe tapering of corticosteroids [183]. Case reports also describe its successful use as rescue therapy in myasthenic crisis [184].

Zilucoplan, a macrocyclic peptide inhibitor of C5, prevents C5 cleavage and blocks MAC formation. Clinical studies have shown that zilucoplan improves daily functioning in patients with moderate-to-severe generalized AChR-antibody–positive MG, suggesting potential utility even in earlier disease stages [185]. Ravulizumab, another long-acting monoclonal antibody targeting C5, maintains therapeutic serum concentrations that allow dosing every 8 weeks [186]. The phase 3 CHAMPION MG trial confirmed its efficacy and tolerability in adults with generalized AChR-antibody–positive MG [187,188].

C1 esterase inhibitors represent an innovative therapeutic class that blocks activation of the classical complement pathway. These agents have been shown to effectively control attacks of hereditary angioedema due to C1 esterase deficiency [189,190] and are being investigated as alternatives to eculizumab in solid-organ transplantation [191]. Given the role of C1 in MG pathogenesis, C1 esterase inhibitors may represent a promising therapeutic strategy, although no clinical trials in MG are currently underway.

### 3.5. Treatment of CIDP

Current treatment options for CIDP include intravenous and subcutaneous immunoglobulins [192,193], plasmapheresis [194], glucocorticoids [195], and immunosuppressive agents [196]. More recently, additional strategies have shown efficacy, including monoclonal antibodies targeting the neonatal Fc receptor [197,198,199,200] and Bruton’s tyrosine kinase inhibitors [201,202].

In parallel, components of innate immunity are being investigated as therapeutic targets, with the complement system attracting the greatest attention. Riliprubart (SAR445088) is a humanized monoclonal antibody directed against C1s, acting at the proximal step of the classical complement pathway. Selective inhibition of the C1 complex prevents downstream complement activation, thereby potentially mitigating inflammatory processes implicated in CIDP pathogenesis. Compared with distal complement inhibitors such as C5 blockers, its targeted mechanism may provide a more favorable safety profile, particularly with respect to infection risk. To date, riliprubart has demonstrated good tolerability and safety [203,204], and a phase 3 clinical trial is currently underway [205].

Continued investigation of innate immune mechanisms in CIDP pathogenesis holds promise for the development of novel, targeted and effective therapeutic strategies for this disease. A summary of currently used and emerging therapies directed against innate immune components is provided in Table 1.

## 4. Conclusions

Innate immunity, long regarded as merely a nonspecific defense system, is now recognized as significant in the pathogenesis of many immune-mediated neurological diseases. Cells such as microglia, DCs, macrophages, neutrophils, and NK cells not only initiate inflammatory responses but also fine-tune them through selective modulation, in close interplay with adaptive immune mechanisms.

In the disorders analyzed—MS, NMOSD, MOGAD, MG and CIDP—extensive experimental, preclinical and clinical evidence supports the involvement of innate effector mechanisms in both disease initiation and progression. Increasingly detailed characterization of molecular pathways and effector-cell phenotypes underscores their potential as therapeutic targets.

However, important gaps remain. Translation of findings from preclinical models to clinical therapies is still limited, and further research is needed to determine which innate immune pathways are most promising and safe for therapeutic modulation. In particular, questions regarding the selective regulation of effector versus regulatory phenotypes, the long-term consequences of complement inhibition, and the interplay between innate and adaptive responses in chronic disease remain unanswered.

Taken together, these findings suggest that the future of neuroimmunological therapy may rest on integrated strategies that combine modulation of innate immunity with interventions targeting adaptive responses. Continued research into the selective regulation of innate immune cell activity and phenotype holds promise for the development of individualized, effective and safe therapeutic approaches.

## Figures and Tables

**Table 1:** Therapies targeting innate immunity.

Disease	Therapeutic Target	Example Therapies	Development Stage
MS	DCs	CTLA4-Ig, anakinra, tocilizumab, TolDCs, nanoparticles, anti-DEC205-MOG	Clinical and preclinical studies
MS	Microglia	PLX5622	Preclinical studies
MS	Kv1.3 channel (microglia, macrophages)	PADRE-Kv1.3 (vaccine)	Preclinical (EAE model)
NMOSD	Complement (C5)	Eculizumab, ravulizumab	Phase 3 (approved drugs)
NMOSD	Eosinophils	Cetirizine	Preliminary reports (class IV evidence)
MOGAD	—	—	—
MG	IFN-I	Anifrolumab	Approved in SLE; potential in MG
MG	TLRs	Chaperonin-10, M5049, IMO-3100	Phase 1–2a in other autoimmune diseases
MG	Complement (C5)	Eculizumab, ravulizumab, zilucoplan	Approved and used clinically
MG	Complement (C1 esterase)	Conestat alfa	Promising; no active MG trials
CIDP	Complement (C1s)	Riliprubart	Phase 3

## Abbreviations

The following abbreviations are used in this manuscript:

Abbreviation	Full Name
MS	Multiple Sclerosis
NMOSD	Neuromyelitis Optica Spectrum Disorder(s)
MOGAD	Myelin Oligodendrocyte Glycoprotein Antibody-Associated Disease
MG	Myasthenia Gravis
CIDP	Chronic Inflammatory Demyelinating Polyneuropathy
DCs	Dendritic Cells
NK	Natural Killer (cells)
ILCs	Innate Lymphoid Cells
CNS	Central Nervous System
EAE	Experimental Autoimmune Encephalomyelitis
CSF	Cerebrospinal Fluid
BBB	Blood–Brain Barrier
Th17	T helper 17 (cells)
IL-17	Interleukin-17
GM-CSF	Granulocyte–Macrophage Colony-Stimulating Factor
TolDCs	Tolerogenic Dendritic Cells
Tregs	Regulatory T Cells
CTLA-4	Cytotoxic T-Lymphocyte Antigen-4
IFN-γ	Interferon-Gamma
IL-2	Interleukin-2
PAMPs	Pathogen-Associated Molecular Patterns
CXCL12	C-X-C Motif Chemokine Ligand 12
FASL	Fas Ligand
TRAIL	TNF-Related Apoptosis-Inducing Ligand
ADCC	Antibody-Dependent Cellular Cytotoxicity
CDC	Complement-Dependent Cytotoxicity
MAC	Membrane Attack Complex
IgG	Immunoglobulin G
AQP4	Aquaporin-4
AQP4-IgG	Aquaporin-4–Specific Immunoglobulin G
C9neo	Neoantigen of C9 (marker of MAC)
EAAT2	Excitatory Amino Acid Transporter 2
FcγRIII (CD16)	Fc Gamma Receptor III (Cluster of Differentiation 16)
NE	Neutrophil Elastase
CCR3	C-C Chemokine Receptor Type 3
IL-5	Interleukin-5
ECP	Eosinophil Cationic Protein
EDN	Eosinophil-Derived Neurotoxin
EPX	Eosinophil Peroxidase
MBP	Major Basic Protein
TREM2	Triggering Receptor Expressed on Myeloid Cells-2
MOG	Myelin Oligodendrocyte Glycoprotein
MOG-IgG	Myelin Oligodendrocyte Glycoprotein–Specific Immunoglobulin G
ADCP	Antibody-Dependent Cellular Phagocytosis
AChR	Acetylcholine Receptor
TLR	Toll-Like Receptor
PV	Poliovirus
EBV	Epstein–Barr Virus
IFN-β	Interferon-Beta
GC	Germinal Center
IFN-I	Type I Interferons
TECs	Thymic Epithelial Cells
APCs	Antigen-Presenting Cells
NMJ	Neuromuscular Junction
CXCR5	C-X-C Motif Chemokine Receptor 5
Tfh	Follicular Helper T Cells
EAMG	Experimental Autoimmune Myasthenia Gravis
TGF-β1	Transforming Growth Factor-Beta 1
NKT cells	Natural Killer T Cells
α-GalCer	Alpha-Galactosylceramide
Foxp3	Forkhead Box P3
Bcl-2	B-cell Lymphoma 2 (protein)
IL-1β	Interleukin-1 Beta
GBS	Guillain–Barré Syndrome
ICAM-1	Intercellular Adhesion Molecule 1
DMTs	Disease-Modifying Therapies
CTLA4-Ig	Cytotoxic T-Lymphocyte Antigen-4 Fusion Protein
Arc/Arg3.1	Activity-Regulated Cytoskeleton-Associated Protein
CSF1R	Colony-Stimulating Factor 1 Receptor
PADRE-Kv1.3	Vaccine targeting Kv1.3 Potassium Channel with PADRE Epitope
IL-6R	Interleukin-6 Receptor
IVIG	Intravenous Immunoglobulins
SLE	Systemic Lupus Erythematosus
RA	Rheumatoid Arthritis
